# Role of Grey Scale and Color Doppler in the Diagnosis of Portal Hypertension: A Comprehensive Review

**DOI:** 10.7759/cureus.103384

**Published:** 2026-02-10

**Authors:** Satyanarayana Kummari, Abhishek J Arora, Mahipal R

**Affiliations:** 1 Department of Radiodiagnosis, All India Institute of Medical Sciences, Bibinagar, Hyderabad, IND; 2 Department of Radiodiagnosis, MNR Medical College and Hospital, Sangareddy, IND

**Keywords:** cavernous transformation of the portal vein, chronic liver disease (cld), cirrhosis of the liver, clinically significant portal hypertension, intrahepatic portal vein, liver elastography, non cirrhotic portal hypertension, oesophageal varices, portal hypertension, portal vein thrombosis (pvt)

## Abstract

Portal hypertension represents a central haemodynamic abnormality in chronic liver disease and is responsible for major complications such as variceal bleeding, ascites, splenomegaly, hepatorenal dysfunction, and hepatic encephalopathy. Current non-invasive imaging contributes substantially to early diagnosis and follow-up; ultrasound (grey scale and colour Doppler) is the most commonly performed modality due to the availability of real-time haemodynamic monitoring and its ability to detect structural and functional derangements. These modalities allow real-time visualisation of portal venous anatomy and dynamic assessment of flow direction, velocity, and collateral circulation, while also capturing parenchymal alterations that reflect disease severity. Although operator dependence and technical limitations can affect diagnostic accuracy, recent advances, including contrast-enhanced ultrasonography, elastography, tissue harmonic imaging, and AI-assisted flow analysis, have markedly strengthened the non-invasive evaluation of portal hypertension. This narrative review synthesises the current evidence on the anatomical and haemodynamic basis of portal hypertension, the diagnostic role of grey-scale and colour Doppler ultrasound, advanced sonographic tools, and evolving technologies that promise to refine prognostication and therapeutic monitoring.

## Introduction and background

Portal hypertension (PH) is an abnormal increase in portal venous pressure as a result of increased resistance to blood flow through the portal venous system [1]. Architectural distortion of hepatic sinusoids, increased intrahepatic vascular resistance, and splanchnic hyperaemia constitute the major causes of this condition, all of which lead to an increase in portal plasma pressure. These haemodynamic alterations then translate into abnormal liver perfusion and systemic multi-organ failure. Clinically significant portal hypertension (CSPH), defined by a hepatic venous pressure gradient (HVPG) of ≥10 mmHg, carries a substantial risk of gastro-oesophageal varices, life-threatening haemorrhage, ascites, spontaneous bacterial peritonitis, and hepatic encephalopathy [1-3].

The global public health impact of portal hypertension continues to rise, driven primarily by an increasing prevalence of chronic liver diseases, such as alcohol-related cirrhosis, hepatitis B and C, and non-alcoholic fatty liver disease [1-3]. Moreover, schistosomiasis is a major cause in endemic areas, and other significant aetiologies apart from cirrhosis include portal vein thrombosis (PVT) due to hypercoagulable states or secondary to abdominal infection or surgical insult [4, 5]. The overall morbidity and mortality associated with these various causes are high in the global population [1, 6].

It is important that portal hypertension be detected early, as failure to do so can lead to complications such as the development of oesophageal varices, ascites, spontaneous bacterial peritonitis, and hepatic encephalopathy [7]. Early detection enables prompt intervention for portal hypertension through endoscopic surveillance of varices, prophylaxis against variceal haemorrhage, and monitoring in the context of deteriorating liver function. Without population-based screening, some patients remain undiagnosed until they present clinically with an acute event [8].

Non-invasive diagnosis of portal hypertension has become a reliable tool for evaluating this disorder, eliminating the need for invasive techniques such as HVPG measurement. Among these, ultrasound with colour Doppler assessment is the first-line tool that offers the possibility of an in vivo evaluation of both anatomical changes and, more importantly, real-time portal haemodynamic analysis [4]. The same examination, providing information on liver morphology, including portal vein (PV) diameter, flow velocity, and hepatic arterial resistive indices, as well as spleen dimensions and the presence of portosystemic collaterals (PSC), can be used to assess disease severity. Its low cost and ease of transport, without exposure to harmful radiation, support its inclusion as part of regular clinical evaluation and long-term follow-up [4, 9, 10]. This narrative review consolidates the existing evidence on the anatomical and haemodynamic foundations of portal hypertension, the diagnostic functions of grey-scale and colour Doppler ultrasound, advanced sonographic techniques, and emerging technologies that aim to enhance prognostication and treatment oversight.

## Review

Relevant anatomy and haemodynamics of the portal venous system

Structure and Normal Flow Patterns

**Figure 1:**
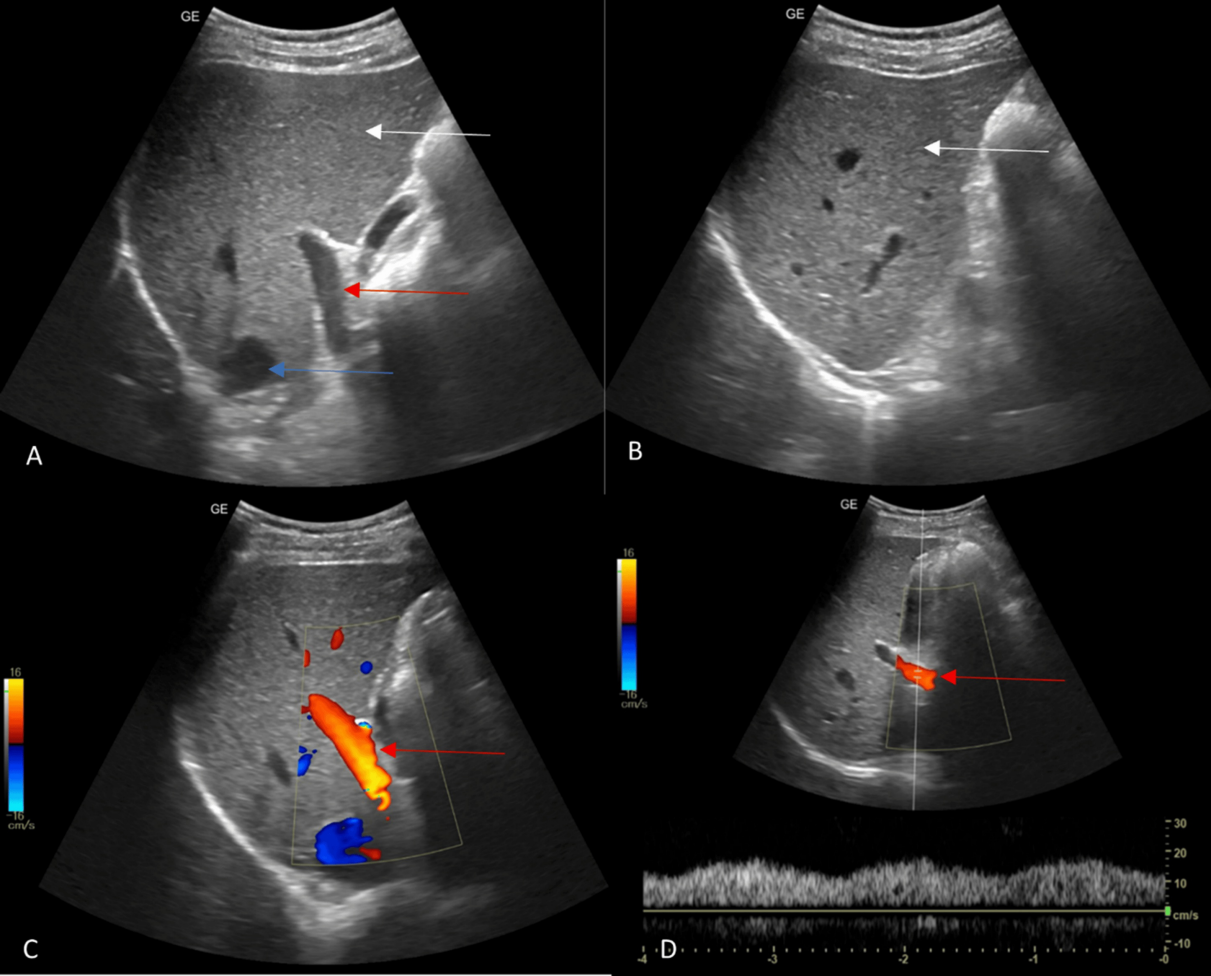
(A and B): Grey scale images of the liver show normal size, echotexture, and smooth surface of the liver (white arrow), normal diameter of the portal vein (red arrow) and IVC (blue arrow). (C and D) Colour and pulsed Doppler images of the portal vein show normal hepatopetal low (red arrows), normal velocity and waveform pattern. The images included in this review manuscript are from our own clinical cases and are used with appropriate permissions

The portal vein system is a low-pressure system with high blood flow that brings nutrient-rich venous blood from the gastrointestinal tract, spleen, and pancreas towards the liver. It is formed by the portal vein, splenic vein, and superior mesenteric vein, which come together to provide two-thirds of the total hepatic blood flow. Under normal conditions, portal venous flow is continuous; a hepatopetal flow direction with low pulsatility mirrors the constant splanchnic inflow. Such stable inflow is also crucial for hepatic metabolic and detoxifying functions (Figure 1) [11].

Physiological Liver Blood Supply and Pressure Differences

The liver has a dual blood supply consisting of the hepatic artery and portal vein, and approximately 70-75% of total liver perfusion is supplied by the portal venous circulation, while the remaining amount is provided by arterial blood. In portal hypertension, diminished portal perfusion triggers the hepatic arterial buffer response, resulting in increased hepatic arterial resistance and compensatory hyperdynamic flow, key Doppler findings [1]. Normal portal pressure is maintained within a range of 5-10 mmHg by a fine-tuned equilibrium between hepatic vascular resistance and splanchnic blood inflow. The gradient between the PV and IVC, the hepatic venous pressure gradient (HVPG), is usually <5 mmHg. This gradient ensures rapid blood exchange between the hepatic sinusoids and the systemic circulation [12].

Haemodynamic Changes in Portal Hypertension

Portal hypertension occurs due to a pathological increase in portal venous pressure, most commonly caused by increased resistance to blood flow within the liver or the portal venous system [8]. Cirrhosis is the most frequent aetiology and results in architectural distortion of the hepatic parenchyma, sinusoidal fibrosis, and regenerative nodule formation, which together increase intrahepatic resistance. In addition, splanchnic vasodilation raises portal inflow and further promotes the pressure load. These haemodynamic alterations lead to the formation of portosystemic collaterals, splenomegaly, ascites, and, in the most severe cases, reversal of portal flow. As abnormal blood flow persists over long periods, liver function is increasingly impaired, and the risk of developing varices and bleeding becomes elevated [8].

Role of conventional ultrasound in portal hypertension evaluation

B-Mode Ultrasound Findings

Grey-scale ultrasound remains the initial imaging modality for assessing suspected portal hypertension. Although it does not directly measure pressure, it provides essential morphological and structural information that correlates strongly with disease severity.

**Figure 2:**
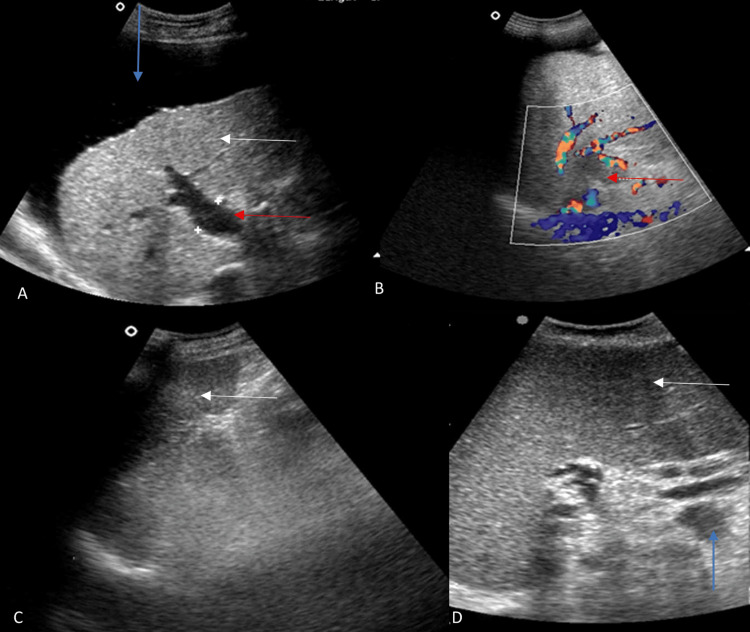
(A) Grey scale image of the liver shows small size, coarse echotexture and surface nodularity of the liver suggesting cirrhosis of liver (white arrow), dilated portal vein (red arrow) and ascites (blue arrow). (B) Colour Doppler image of the portal vein shows no colour flow in the portal vein suggesting portal vein thrombosis (red arrow). (C and D) Grey scale images of the spleen show enlarged spleen suggesting splenomegaly (white arrows) and multiple splenic hilar collaterals (blue arrow). The images included in this review manuscript are from our own clinical cases and are used with appropriate permissions

Liver morphology: Cirrhotic transformation results in coarse parenchymal echotexture, nodular surface irregularity, and alterations in lobar architecture, such as hypertrophy of the caudate and left lobes with relative right lobe atrophy. These changes, while not pathognomonic, are highly suggestive of chronic liver disease and serve as indirect markers of portal hypertension (Figure 2) [9].

Portal vein diameter: Portal vein diameter is often assessed by B-mode ultrasound and varies in adults from 10 to 13 mm in healthy individuals. Dilatation can be an early marker of elevated portal pressure, but the size as such does not meet diagnostic accuracy criteria, primarily because it overlaps with normal variants. A portal vein diameter exceeding 13-15 mm is a widely accepted sonographic indicator of portal hypertension. This enlargement reflects chronic pressure elevation and reduced vascular compliance [13, 14].

Splenomegaly: Splenomegaly (size >12 cm in the longitudinal axis) is a common architectural alteration in portal hypertension. Splenomegaly can be precisely demonstrated by B-mode ultrasound, which, due to hepatosplenic venous pressure rises and high splenic capillary retention, causes blood to accumulate in the reticulo-endothelial system [15].

Ascites: Ascites is evidence of decompensated portal hypertension and represents the presence of free intraperitoneal fluid that can be easily demonstrated using grey-scale imaging. Clinicians can assess the severity of disease and the likelihood of complications by identifying even small fluid collections. Sonographic features such as internal echoes or septations may suggest infection or malignancy [16].

Collateral circulation: B-mode imaging is useful for demonstrating dilated, tortuous collateral veins (e.g., paraumbilical, splenic, and gastro-oesophageal collaterals) secondary to increased portal pressure, with flow rerouted through other vessels [17].

Portal venous system evaluation: Grey-scale ultrasound aids in identifying portal vein thrombosis, cavernous transformation, and extrinsic compression. An acute thrombus appears echogenic, while chronic thrombosis may demonstrate multiple serpiginous channels representing collateral formation. Although grey-scale findings alone cannot confirm portal hypertension, they provide essential context that complements Doppler-derived haemodynamic measurements [13, 14].

Ultrasound-Based Grading Systems

Congestive index: The congestive index, defined as the PV cross-sectional area divided by the mean flow velocity, is a semiquantitative index of PV haemodynamics. Larger values are indicative of increased portal hypertension and poorer venous return [10, 18].

Parenchymal echotexture scoring: Liver parenchymal echotexture is scored based on changes in texture, such as coarsening, nodularity, and heterogeneity, which are indicative of chronic liver disease. The aspartate transaminase (AST) to platelet ratio index (APRI) is a non-invasive biochemical index used to assess structural morphological changes in the liver, particularly fibrosis and cirrhosis, without biopsy. A high APRI is associated with a higher stage of fibrosis and an increased risk of portal hypertension [19].

Limitations of Grey-Scale Ultrasound

Operator dependency: There is significant variation in ultrasound results between highly skilled and average sonographers, as reported in interobserver studies comparing measurements and interpretations [20].

Reduced sensitivity in early disease: Early or compensated portal hypertension is not associated with structural alterations of the arterial and venous circulation, and the sensitivity of B-mode ultrasound at this stage is low, as splenomegaly or shunt development may not have occurred [21].

Body habitus and bowel gas interference: Obese patients, overlying bowel gas, and difficult acoustic windows may reduce image quality, which adversely affects B-mode reliability and may necessitate further Doppler evaluation for more reliable assessment [22].

Role of colour Doppler ultrasound in portal hypertension

Colour and spectral Doppler represent the cornerstone of non-invasive assessment of portal hypertension. They provide dynamic, quantitative information regarding vascular flow patterns that closely mirror underlying physiologic alterations.

Portal Vein Flow Velocity

The velocity of blood flow in the portal vein is one of the valuable Doppler indexes for probing portal haemodynamics. Mean flow velocity is typically 15-20 cm/s in normal adults. In portal hypertension, however, this velocity is often reduced secondary to increased intrahepatic resistance and abnormal splanchnic haemodynamics. Of note, in previous studies, a flow velocity inferior to 12 cm/s has been reported to be indicative of clinically relevant portal hypertension, and much lower values may suggest severely reduced portal flow [23].

Flow Direction Abnormalities

Hepatopetal to hepatofugal reversal: Hepatic portal flow is usually hepatopetal, directed toward the liver. This can be readily observed because increased pressure within the portal venous system causes blood to be diverted away from the liver into portosystemic collateral pathways, with flow directed caudally (toward the feet), resulting in reversal of portal venous flow [24].

Biphasic or turbulent flow: In severe cases, turbulent, bidirectional, or biphasic portal venous flow may be observed, indicating markedly deranged haemodynamics and increased intrahepatic resistance within the portal system. These flow abnormalities are strongly associated with the progression and severity of the disease [24].

Portal Vein Congestion Index (CI)

The congestion index, calculated as the ratio of the portal vein cross-sectional area to mean flow velocity, serves as a reliable marker of portal flow stagnation. Normal values range from 0.07 to 0.10, while a CI exceeding 0.15-0.20 is strongly predictive of portal hypertension. This index is particularly valuable in scenarios where the portal vein is markedly dilated, accompanied by sluggish flow [11, 18].

Portal Venous Pulsatility Index (PI)

A blunted waveform with reduced pulsatility often indicates increased intrahepatic stiffness. Conversely, exaggerated pulsatility may reflect cardiac causes of portal hypertension [11, 18, 23].

Hepatic Artery Resistive Index (HARI)

Doppler indices of the hepatic artery provide additional information on resistance within the hepatic vasculature. The hepatic artery RI in normal individuals ranges from 0.55 to 0.70 but increases in portal hypertensive conditions in response to compensatory arterial vasoconstriction and sinusoidal compression, with higher RI and PI values related to advanced grades of portal hypertension and cirrhotic remodelling. The HARI increases as portal venous inflow decreases, a compensatory mechanism driven by the hepatic arterial buffer response. Values exceeding 0.78-0.80 suggest advanced portal hypertension. This Doppler parameter also correlates with fibrosis stage and early decompensation [25].

Splenic Artery Resistive Index (RI)

Because splenic resistance increases with congestion, an RI >0.65-0.70 suggests chronic portal hypertension and has prognostic value for variceal formation [7, 23].

Hepatic Vein Waveform Index

Loss of the normal triphasic hepatic venous waveform indicates increased parenchymal stiffness, loss of hepatic compliance, and advanced fibrosis or cirrhosis. The progressive transition from triphasic to biphasic and then monophasic patterns correlates with increasing disease severity [7, 23].

Splenic and Superior Mesenteric Venous Flow

The splenic vein commonly exhibits dilatation (>10 mm) and reduced flow velocity in portal hypertension. Altered haemodynamics in the superior mesenteric vein may similarly reflect increased splanchnic resistance or collateralisation [26, 27].

Doppler Assessment of Portosystemic Collaterals

Colour Doppler excels at detecting spontaneous portosystemic collaterals. Common pathways are as follows.

Paraumbilical vein: Recanalisation and dilatation of the paraumbilical vein are often concomitant findings in long-standing cases of portal hypertension. Colour Doppler reveals continuous hepatofugal flow, consistent with severe portosystemic shunting [26, 27].

Left gastric (coronary) vein: The left gastric vein is often significantly dilated in patients with oesophago-gastric varices. A colour Doppler image depicting hepatofugal flow in this vein is a high-grade predictor of variceal formation and haemorrhage [28].

Splenorenal and gastrorenal shunts: Splenorenal shunts are vascular connections between the splenic vein and the left renal vein, allowing portal blood to bypass the liver. Gastrorenal shunts occur between the gastric veins, usually the left gastric or short gastric veins, and the left renal vein [26-28].

Perisplenic, perigastric, and gallbladder wall collaterals: Perisplenic collaterals arise from dilatation of venous channels around the splenic hilum and capsule and are commonly associated with splenomegaly and hypersplenism, reflecting long-standing elevation of portal pressure. Perigastric collaterals primarily develop along the lesser curvature and fundus of the stomach, originating from the left and short gastric veins; these collaterals form the haemodynamic basis for oesophageal and gastric varices, which increase the risk of upper gastrointestinal bleeding. Gallbladder wall collaterals, also referred to as portal hypertensive cholecystopathy, occur due to diversion of portal flow through cystic and pericholecystic veins and manifest as serpiginous vascular channels within the gallbladder wall, which may mimic inflammatory wall thickening on imaging and pose a risk of bleeding during surgical intervention [27-28].

Colour Doppler in Portal Vein Thrombosis (PVT)

Bland vs. malignant thrombus discrimination: Colour Doppler is useful for thrombus distinction. Bland thrombi are hypovascular, whereas malignant thrombi, more frequently seen in hepatocellular carcinoma, present with intra-thrombus arterial or chaotic flow [10, 29].

Flow gaps and cavernous transformation: Chronic PVT may result in cavernous transformation and is responsible for the formation of multiple serpiginous collaterals that replace the normal portal vein. The presence of these meandering vessels can be identified on colour Doppler, along with the relative absence of blood flow between them, indicating their chronic nature and impact on portal haemodynamics [30].

Advanced Doppler techniques

Power Doppler Imaging

Power Doppler is more sensitive than conventional colour Doppler for identifying slow and low-volume flow, which is common in advanced portal hypertension. Power Doppler enhances visualisation of subtle vascular structures by displaying Doppler signal amplitude rather than velocity or direction. It excels at detecting pericholecystic and perisplenic collaterals, cavernous transformation of the portal vein, reticular collateral channels in the porta hepatis, and diminutive hepatofugal pathways that are challenging to identify with standard Doppler. This modality is particularly valuable in patients with chronic thrombosis or extensive collateral networks [31].

Tissue Harmonic Imaging (THI)

Tissue Harmonic Imaging improves image quality through reduction of noise and artefacts, resulting in better images in patients with difficult acoustic windows, such as obese or ascitic patients or those with overlying bowel gas. Vascular anatomy, liver surface nodularity, and parenchymal abnormalities are better visualised with THI, enabling a more accurate assessment of the portal venous system and its associated tissues [32].

Contrast-Enhanced Ultrasound (CEUS)

CEUS uses microbubble contrast agents to augment vascular imaging and offers real-time assessment of microcirculation and portal venous enhancement dynamics. CEUS has key advantages in portal hypertension imaging. It excels at detecting portal vein thrombosis, as bland thrombus shows no internal enhancement, whereas tumour thrombus exhibits arterial-phase enhancement. It also allows visualisation of slow collateral flow that may be invisible on standard Doppler and assessment of intrahepatic perfusion heterogeneity typical of cirrhosis and portal hypertension. As a radiation-free modality without nephrotoxic contrast, CEUS is well suited for patients with renal impairment or contrast allergies [33].

Three-Dimensional (3D) and Four-Dimensional (4D) Doppler

Three-dimensional Doppler facilitates volumetric quantification of flow patterns and mapping of collateral pathways, aiding determination of collateral network volume, visualisation of twisted or tortuous venous pathways, and more accurate assessment of splenorenal and gastrorenal shunts. Although not yet widely used clinically, 3D and 4D Doppler techniques hold promise for increased clinical adoption as advancing software improves speed and reproducibility [29, 33].

Elastography (Shear Wave, Transient, and 2D Elastography)

Elastography has transformed non-invasive assessment of portal hypertension by quantifying liver and spleen stiffness. Key diagnostic thresholds include liver stiffness ≥15 kPa, suggestive of CSPH, liver stiffness ≥20-25 kPa, strongly predictive of varices, and spleen stiffness >46-55 kPa, which correlates with high-risk varices and CSPH. Spleen stiffness, reflecting splenic congestion, is increasingly recognised as a superior predictor of variceal risk compared with liver stiffness alone. Combining spleen stiffness with portal vein velocity and left gastric vein diameter yields excellent diagnostic performance [34].

Comparison of diagnostic performance

Ultrasound/Doppler vs. Computed Tomography (CT), Magnetic Resonance Imaging (MRI), and HVPG

To date, ultrasound with colour Doppler remains the main modality for studying portal hypertension due to its wide accessibility, safety, and ability to provide instant haemodynamic evaluation. CT and MRI, however, offer better anatomic resolution and are preferable for deep collateral imaging, characterisation of liver morphology, and identification of vascular pathologies such as portal vein thrombosis or cavernoma formation. However, these methods are unable to evaluate flow dynamics in the manner provided by Doppler imaging [35].

The HVPG remains the reference standard for detection and staging of portal hypertension. An HVPG >10 mmHg represents clinically significant portal hypertension, and a value >12 mmHg indicates an increased risk of variceal haemorrhage. Because HVPG measurement is invasive and costly, Doppler ultrasound, although less reliable than HVPG, is considered a useful non-invasive tool for screening and monitoring [35].

Sensitivity and Specificity of the Doppler Markers

Doppler ultrasound findings, including reduced portal vein velocity, hepatofugal flow, increased hepatic artery resistive index, and dilated collaterals, strongly predict a diagnosis of portal hypertension with good to excellent diagnostic accuracy. A low peak systolic velocity (PSV) (<12-13 cm/s) of portal flow appears to be fairly sensitive and specific for significant portal hypertension. Hepatofugal flow is highly specific for cirrhosis but is much less sensitive in early disease [36].

Collateral flow demonstrated by Doppler is highly diagnostic. Paraumbilical and left gastric vein flow alterations are very specific for varices. However, inter-operator variability and sensitivity to body habitus remain concerns, particularly when disease is mild or compensated. In summary, while Doppler markers cannot replace HVPG, their combined use offers a non-invasive tool with excellent diagnostic accuracy [31].

Sonographic markers of clinically significant portal hypertension

Although ultrasound cannot directly quantify pressure, certain combinations of findings strongly predict clinically significant portal hypertension (CSPH; HVPG >10 mmHg). Highly predictive markers include portal vein velocity <12 cm/s, portal vein diameter >13-15 mm, persistent hepatofugal flow, loss of respiratory phasicity, spleen length >13-15 cm, splenic vein dilatation (>10 mm), recanalised umbilical vein, left gastric vein diameter ≥6 mm, liver stiffness >15 kPa, splenic stiffness >46 kPa, and hepatic artery RI >0.78.

Composite algorithms combining low portal vein velocity, splenomegaly, and the presence of collaterals achieve some of the highest non-invasive predictive accuracies for clinically significant portal hypertension [14-15, 23, 25, 27, 34, 36].

Role of ultrasound in monitoring progression and treatment response

Screening of Oesophageal Varices and Rebleeding Risk

While endoscopy remains the current gold standard for diagnosing oesophageal varices, the information derived from colour Doppler ultrasound may aid in less invasive patient screening. Dilatation of the left gastric vein, hepatofugal flow, and paraumbilical collaterals are all strong Doppler predictors of variceal formation and bleeding. These markers do not, however, allow stratification of patients who may require shorter intervals to follow-up endoscopy or early prophylactic therapy [37].

Enhanced Flow Velocity With Beta-Blockers

Non-selective beta-blockers decrease portal pressure by reducing splanchnic outflow. Doppler ultrasound can monitor response to therapy, with a rise in the velocity of flow in the portal vein and reduction in collaterals. The beneficial haemodynamic response and reduction in variceal bleeding are associated with the demonstrated enhancement of portal flow velocity following treatment. Therefore, Doppler profiling is an appropriate and non-invasive assay for monitoring of pharmacological activity [38].

Monitoring After Transjugular Intrahepatic Portosystemic Shunt (TIPS) Placement

Transjugular intrahepatic portosystemic shunt (TIPS) insertion exerts a drastic influence on portal haemodynamics, and Doppler ultrasound has become the first-choice modality to monitor TIPS patients. Relevant parameters are shunt patency, flow velocity in the stent, and the presence of stenosis or occlusion. Under physiological conditions, TIPS flow is in a high-velocity continuous flow pattern; a decrease in linear velocity or the presence of turbulent signals could be associated with future dysfunction. It is possible to intervene while still subclinical using colour Doppler monitoring [39].

Surveillance in Non-cirrhotic Portal Hypertension (NCPH)

Ultrasound has a relevant role in long-term surveillance of patients with NCPH, including portal vein thrombosis, idiopathic portal hypertension, and schistosomiasis. Doppler evaluation is effective in detecting reversal of portal flow, advancement of cavernous transformation, changes in the splenic vein, splanchnic collaterals, or development of new collaterals. Regular follow-up is necessary to identify disease status, the risk of complications, and the need for therapeutic change [40].

Clinical applications and case-based evidence

Cirrhosis

The most frequent cause of portal hypertension is liver cirrhosis, and Doppler ultrasound plays a key role in its evaluation. Features include decreased portal vein flow velocity, elevated hepatic artery resistive index, splenomegaly, and collaterals. On case-based examinations, reversal of portal flow and paraumbilical vein recanalisation are typical findings in end-stage cirrhosis, indicating a higher likelihood of variceal bleeding. These Doppler markers assist in guiding surveillance, medical therapy, and transplant assessment [8, 19].

Portal Vein Thrombosis

The use of colour Doppler ultrasound is essential in the diagnosis of PVT. Acute PVT typically demonstrates echogenic thrombus with absent or reduced flow, whereas chronic PVT appears as cavernous transformation with multiple tortuous collaterals. Doppler differentiates between bland and malignant thrombi using internal arterial signals or neovascularity. It has been strongly endorsed by case series for its ability to follow thrombus resolution and guide anticoagulation therapy [10, 29].

Budd-Chiari Syndrome

In Budd-Chiari syndrome, Doppler ultrasound shows hepatic vein obstruction with absence or reversal of flow and intrahepatic venous collaterals. It is also pertinent in demonstrating caudate lobe hypertrophy and hepatocyte regeneration with compensatory hepatic arterial flow. Case descriptions have shown the sensitivity of Doppler in early detection of hepatic outflow obstruction, allowing anticoagulation or angioplasty to be initiated before irreversible liver damage occurs [41].

Paediatric Portal Hypertension

In children, portal hypertension commonly results from congenital malformations or anomalies, extrahepatic portal vein obstruction present from early life, and only rarely from chronic liver disease. The primary modality of choice for investigation is Doppler ultrasound, which is a safe tool that can evaluate both vascular patency and splenic size without ionising radiation exposure. In a case-based approach, portal cavernoma, splenic varices, and early changes in flow direction can be demonstrated with Doppler, and such information is critical when planning shunt surgery or endotherapeutic interventions [42].

Schistosomiasis-Related Portal Hypertension

Schistosomiasis is a leading cause of NCPH in endemic countries. Disease-related changes, including increased portal venous resistance, microscopic periportal fibrosis, and splenic enlargement, are noted on Doppler ultrasound. With disease advancement, hepatopetal flow may be reduced or reversed, and collateral veins may dilate. Clinical descriptions conclude that Doppler may be valuable for grading periportal fibrosis, predicting variceal risk, and monitoring therapy [43].

Limitations and challenges

Early Disease Detection Issues

An early diagnosis of portosystemic flow is a drawback of ultrasound and colour Doppler assessments. Late in the disease course, long-standing haemodynamic alterations can lead to structural changes, manifesting as marked splenomegaly, portal vein dilatation, and formation of collateral vessels on imaging, which may be overlooked or misinterpreted. Early haemodynamics may not yield clear Doppler observations, which could hinder the detection of subclinical portal hypertension [44].

Inter-operator Variability

Operator-dependent variability of ultrasound techniques may be a limitation regarding reproducibility between different operators, as assessed by portal vein velocity and hepatic artery resistive index, as well as identification of portosystemic collaterals. This heterogeneity affects diagnosis and treatability across centres and physicians. In accordance with protocol-based imaging, reliability is increased during routine use and standardised training, which allows improved reproducibility [20, 45].

Lack of Standardised Doppler Cut-Offs

Although guidelines from high-income countries aid interpretation of Doppler indices, there are no internationally accepted cut-off values for abnormality, and population-specific normal reference values need to be defined for low- and middle-income country settings [23, 44].

Even though studies exist in the literature on this theme, to date no Doppler parameters have proven to universally define cut-off values for the diagnosis of clinically significant portal hypertension. In normal individuals and patients with mild or compensated disease, there is substantial overlap in portal vein velocity, hepatic artery RI, and congestion index. This threshold heterogeneity adds an element of variability in interpretation, impairing diagnostic certainty, especially closer to the boundary [23, 44].

The Obese or Ascitic Patients

Obesity, significant subcutaneous fat, and gross ascites greatly influence image quality by making it difficult to distinguish important vascular structures. Bowel gas interference further decreases visibility, resulting in challenging or even poor Doppler examinations. The use of additional imaging modalities, such as CT, MRI, and elastography, should also be considered in these patients [46].

Future directions

Artificial Intelligence (AI): Augmented Doppler Analytics

The AI implementation of Doppler ultrasound is about to change the landscape of portal hypertension assessment. AI-based algorithms can support automatic measurement of portal vein velocity, hepatic artery indices, and collateral flow patterns, decrease operator dependence, and enhance diagnostic consistency. These systems could also assist in earlier detection of subtle haemodynamic changes when compared to routine human interpretation [47].

Quantitative Doppler Biomarkers

A further area in which additional research is required is the validation of these novel Varices Risk Index (VRI) algorithms as surrogate markers of portal venous pressure (PVP) and their comparison with integrated commercial Doppler probes (such as MAR-4), as current evidence is largely extrapolated from heart failure studies and direct patient comparisons are limited. With normalised flow indices (FI), shear-wave-assisted Doppler measurements, and perfusion-based metrics, accuracy levels for identification of clinically significant portal hypertension could be greatly improved. Definition of validated cut-offs of biomarkers would facilitate diagnosis in various clinical contexts [23].

Machine-Learning-Based Flow Classification

Machine learning algorithms trained on large data sets of Doppler flow images have the potential to correctly identify flow patterns (hepatopetal, hepatofugal, biphasic, or turbulent) with better accuracy than visual determination. By associating flow signatures with clinical end points, such models can be used to forecast disease development, variceal risk, and treatment outcome. Automatic classification might promote a standardised reporting system and help clinicians in early decision-making [48].

Integration With 3D/4D Ultrasound

The 3D and 4D ultrasound techniques improve portal vein anatomy visualisation and flow dynamics. Their fusion with Doppler imaging may allow volume flow quantification, improve collateral visualisation, and more accurately assess TIPS function. These advancements, when they become more widely available, could alter the landscape of non-invasive monitoring approaches to portal hypertension [49].

Automated Portal-Flow Mapping

Computer software that could automatically create portal-flow maps in real time could greatly facilitate evaluation. These AI-powered Doppler signal processing approaches have contributed towards producing visual representations of portal haemodynamics and have led to more accurate identification of abnormal flow and areas populated with collateralisation. This type of automation could improve both diagnostic accuracy and workflow in high-throughput hepatology units [50].

## Conclusions

Ultrasound and colour Doppler are imperative non-invasive first-line imaging investigations to assess PH in real time by demonstrating the anatomy and flow dynamics of the PV and the complications associated with it. Their availability and low cost, as well as their capability to explore major haemodynamic changes (diminished portal flow velocity, flow reversal, and development of collaterals), are essential for clinical practice. The combination of the above-mentioned findings with sophisticated ultrasound techniques, including elastography, CEUS, power Doppler, and THI, raises the overall diagnostic accuracy and predictive value of ultrasound and colour Doppler assessments substantially.

Novel technological developments, such as AI-based Doppler analytics, machine learning-based flow classification, and automated portal-flow mapping, present valuable evidence to help overcome current drawbacks, such as operator dependence and variation in Doppler interpretation. Further development of these methods is likely to harmonise evaluation, increase quantitative accuracy, and consolidate ultrasonography as an essential component in the comprehensive diagnosis of portal hypertension.
